# Association between psychoeducational factors and perceived academic stress in medical students: a gender-based analysis

**DOI:** 10.1186/s12909-026-08770-2

**Published:** 2026-02-10

**Authors:** Carolina Lagares-Franco, María Jesús Viñolo Gil, Cristina O´Ferrall González, Horacio López Ruiz, Ismael García-Campanario

**Affiliations:** 1 Institute for Biomedical Research and Innovation of Cádiz, Cadiz, 11009 Spain; 2 Department of Statistics and Operational Research, Faculty of Medicine, University of Cadiz, Cadiz, 11003 Spain; 3 Research Group PAIDI UCA CTS553, University of Cadiz, Cadiz, 11003 Spain; 4 Department of Nursing and Physiotherapy, University of Cadiz, Cadiz, 11003 Spain; 5 Rehabilitation Clinical Management Unit, Interlevels-Intercenters Hospital Puerta del Mar, Hospital Puerto Real, Cadiz Bay-La Janda Health District, Cadiz, 11006 Spain; 6 Research Group PAIDI UCA CTS1152, University of Cadiz, Cadiz, 11003 Spain; 7 Department of Medicine and Surgery, Faculty of Medicine, University of Cadiz, Cadiz, 11003 Spain; 8 Research Group PAIDI UCA CTS391, University of Cadiz, Cadiz, 11003 Spain; 9 Servicio de Urgencias de Atención, Primaria Hospital Universitario de Jerez de la Frontera, Cádiz, España

**Keywords:** Academic stress, Medical education, Sleep quality, Physical activity, Gender difference

## Abstract

**Background:**

Academic stress is a dynamic cognitive appraisal process in which students perceive educational demands as exceeding their coping resources This perception is associated with emotional and behavioral responses that relate to well-being and perceived academic stress.

**Objective:**

This study aimed to identify the main academic stressors in medical students and to examine their relationship with sleep quality, physical activity, and gender, specifically focusing on perceived stress rather than academic grades.

**Methods:**

An analytical cross-sectional study was conducted including sociodemographic questions and validated instruments: the Pittsburgh Sleep Quality Index (PSQI), the Academic Stress Questionnaire (E-CEA), and the short form of the International Physical Activity Questionnaire (IPAQ). Logistic regression analyses were performed.

**Results:**

Significant gender-related differences were found in stress responses and coping strategies. Female students reported higher levels of academic overload (*p* = 0.004), emotional demands, and exam-related anxiety (*p* = 0.005), as well as differences in exam preparation strategies (*p* = 0.037). Regarding lifestyle factors, poor sleep quality was identified as a significant factor associated with higher stress levels (OR = 1.12; 95% CI: 1.03–1.22).

**Conclusion:**

Academic overload and exam anxiety were identified as significant factors associated with medical students´ well-being. Female students showed a higher probability of reporting stress (OR = 5.56). These findings highlight the need for gender-sensitive psychoeducational interventions that promote healthy sleep habits and stress management alongside physical activity recommendations.

**Supplementary Information:**

The online version contains supplementary material available at 10.1186/s12909-026-08770-2.

## Introduction

Research shows that health sciences students experience higher stress levels than students in other fields, tending to increase in later years, especially when students are immersed in clinical settings [1, 2]. Added to these academic conditions are contextual factors, such as socio-family and sociodemographic variables, which are significantly associated with the experience of academic stress. For instance, first-generation students often report higher stress levels compared to those with college-educated parents [3, 4]. Furthermore, living arrangements play a role; students who live with their families tend to experience lower stress levels than those living independently [5].

Academic stress continues to be a global problem in the university population since most preventive interventions have limited long-term effectiveness [6]. Structural sources of stress include concerns about the development of professional skills, the ability to adapt to the future, academic load, and financial hardship, all of which emerge as critical factors associated with student well-being [7].

The analysis of academic stress combined with a gender approach makes it possible to visualize significant differences in the perception of academic load, self-confidence, and coping strategies [8]. Likewise, female students often report greater impairment of sleep and emotional regulation [9], which shows a strong association with their perceived academic stress. Therefore, designing gender-sensitive psychoeducational interventions is necessary to promote emotional well-being [10, 11]. Doctors are vulnerable at all stages of their careers, recognizing this risk, we propose that upstream strategies aimed at building resilience should be created, including targeted prevention for medical students [12].

The stress response involves the relationship between the subject and their environment, where environmental demands are perceived as exceeding the individual´s coping resources [13]. For university students, managing a demanding academic schedule, coupled with the high frequency of assessments and uncertainty about evaluation methods, creates significant stressors that related to both physical and mental health [14]. These challenges also reduce leisure time and physical activity, contributing to sleep disorders, which are more common during exam periods [15, 16].

In this context, the present study analyzes the psychoeducational factors associated with academic stress and sleep quality in medical students, considering gender differences and the modulating role of physical activity as a coping strategy.

In line with previous evidence and the need for specific interventions, the specific aim was to identify the main academic stressors affecting medical students at the University of Cadiz, as well as to analyze gender differences in perceived academic stress, physical activity, and sleep quality. Our null hypothesis was that there were no significant differences in academic stress levels with respect to gender, sleep quality, and physical activity.

## Materials and methods

### Study design and participants

An observational, analytical cross-sectional study was carried out among medical students at the University of Cadiz. Data collection was conducted between October and November 2023, during a regular academic period free of final examinations. This timeframe was selected to minimize the confounding effect of acute exam-related anxiety, allowing for a more accurate assessment of baseline stress levels and typical lifestyle habits. Participation was completely voluntary and anonymous. All undergraduate students were invited to participate through institutional email. Those randomly selected were informed of their right to participate or refuse without consequences.

### Sample size calculation

Based on a survey carried out by the State Council of Medical Students (CEEM) and the Spanish Association of Medical Education (SEDEM), carried out in 43 medical schools in Spain, it was found that 36.8% of students had academic stress [17]. Based on this percentage, a minimum sample size of 185 students was estimated, considering a margin of error of 6.6% and a significance level of 5%.

### Inclusion and exclusion criteria

To be included in the study, participants had to: (i) be of legal age, (ii) be exclusively enrolled in the study program (non-Erasmus exchange students), and (iii) provide informed consent. Exchange students were excluded because they were easily identifiable and to ensure cultural and linguistic homogeneity in the perception of local academic stressors.

It is important to note that the gender distribution of the sample (78% female) mirrors the current demographic reality of medical students at the University of Cadiz and reflects the broader trend of the feminization of medicine in Spain [18], ensuring the ecological validity of the findings regarding gender differences.

### Data collection

The data were collected through an online survey developed in Google Forms. The survey was designed to collect sociodemographic data, including age, gender, year of study (1st to 6th grade), whether the student received financial aid or scholarship (yes/no), living situation during the year of study (living alone, with parents or guardians, in a student flat, in a shared flat or other) and whether the student received social support from the family (yes/no). Upon completion, the survey responses were exported to an Excel document for efficient coding and data management. The survey link was distributed to students through class representatives, ensuring wide accessibility.

### Assessment instruments

#### Level of physical activity

It was assessed using the International Physical Activity Questionnaire (IPAQ) in its abbreviated version. This questionnaire has been validated for Spanish adults aged 18 to 69 years with a reliability coefficient equal to 0.65 [19]. It consists of seven items that collect information on the frequency, duration and intensity of physical activities carried out in the seven days prior to the assessment. Weekly physical activity is quantified using equivalent metabolic task units (METs) per minute per week. Light activities such as walking are assigned a value of 3.3 METs, moderate activities are assigned 4 METs, and vigorous activities are assigned 8 METs. The total MET score is calculated by multiplying the baseline by the daily time spent on the activity and the number of days per week the activity is performed.

#### Academic stress

The “Academic Stress Questionnaire” (E-CEA) [20] was used. It consists of 54 items grouped into eight factors (e.g., Academic Overload, Exam, Methodology) evaluated on a 5-point Likert scale (1 = never to 5 = always). This final score represents the stress potential for the purpose of the logistic regression analyses, the variable was dichotomized based on the instrument’s validation guidelines: a mean score of ≥ 3 was established as the cut-off point to distinguish “High Stress” (clinically significant) from “No/Low Stress”. The instrument has shown high reliability in Spanish students (Cronbach’s alpha = 0.96).

#### Sleep quality

The Pittsburgh Sleep Quality Index (PSQI) was applied [21]. It includes 19 items assessing seven components of sleep. A global score > 5 was used as a cut-off point to identify poor sleepers. This questionnaire has been validated in Spanish students (reliability coefficient = 0.81).

### Statistical analysis

A descriptive and inferential analysis was performed to summarize the main characteristics of the data, with special attention to quantitative and qualitative variables. This analysis involved the calculation of the mean and standard deviation of the quantitative variables, providing measures of central tendency (mean and median) and dispersion (standard, maximum, and minimum deviation). For the qualitative variables, absolute frequencies and percentages were obtained. The relationship between qualitative variables was evaluated using the chi-square test. Normality was assessed using the Kolmogorov-Smirnov test. When the sample followed a normal distribution, contrast tests based on Student’s T-distribution were applied, while Wilcoxon’s test was used in cases where normality could not be assumed. To examine the relationship between academic stress and the different factors studied, multivariate logistic regression models were applied. Significant or near-significant variables (*p* < 0.1) in the bivariate analysis were added using the introduction method and those whose results were not significant in the model were discarded. The Hosmer-Lemeshow test was used to test goodness of fit and the Wald statistic for coefficients. In all cases, a significance level of 5% was considered and the analysis was carried out with the IBM SPSS v.26 program.

### Ethical considerations

The study adhered to the ethical standards described in the Declaration of Helsinki and received approval from the Committee on Ethics and Non-Biomedical Research (CEENBOMGs) of the University of Cadiz (Ref. 010_2023). Informed consent was obtained from all participants, and data confidentiality was guaranteed throughout the process.

## Results

The sample consisted of 187 students, of which 78% were women. The overall mean age was 22.34 years (SD = 5.12). The average age was 21.92 years (SD = 3.78) for women and 23.78 years (SD = 8.16) for men, with no statistically significant difference (*p* = 0.164). Regarding sociodemographic characteristics, the majority were first-year students, 35.3% received scholarships, and 88% lived with their parents or in shared housing, A history of mental illness was reported by 19.25% of participants. Regarding support systems, (91.7%) reported feeling supported by their family and 8.1% reported other forms of social support (Table 1).

**Table 1:** Sample population characteristics

		Women *n* (%)	Men *n* (%)	Total *n* (%)
Academic Year	1st	27 (18.5)	15 (36.6)	42 (22.5)
	2nd	26 (17.8)	1 (2.4)	27 (14.4)
	3rd	29 (19.9)	7 (17.1)	36 (19.3)
	4th	27 (18.5)	6 (14.6)	33 (17.6)
	5th	21 (14.4)	4 (9.8)	25 (13.4)
	6th	16 (11.0)	8 (19.5)	24 (12.8)
Scholarship recipient	Yes	46 (31.5)	20 (48.8)	66 (35.3)
	No	100 (68.5)	21 (51.2)	121 (64.7)
Type of Residence	Housing Familiar	64 (43.8)	22 (53.7)	86 (46.0)
	Shared apartment	67 (45.9)	12 (29.3)	79 (42.2)
	Residence University	6 (4.1)	2 (4.9)	8 (4.3)
	Independent	3 (2.1)	4 (9.8)	7 (3.7)
	Other	6 (4.1)	1 (2.4)	7 (3.7)
History of mental illness	Yes	29 (19.9)	7 (17.1)	36 (19.3)
	No	117 (80.1)	34 (82.9)	151 (80.7)
Family Support	Yes	130 (90.9)	36 (94.7)	166 (91.7)
	No	13 (9.1)	2 (5.3)	15 (8.3)
Social support	Yes	119 (85.0)	36 (90.0)	155 (86.1)
	No	21 (15.0)	4 (10.0)	25 (13.9)

Values are presented as frequency (*n*) and percentage (%)

The mean score on the Academic Stress Questionnaire (E-CEA) was 105.74 points (SD = 32.7) Women scored higher on average (108.9 ± 30.9) than men (94.3 ± 36.6). when analyzing the dimensions of the questionnaire based on high or low stress potential, gender and sleep quality emerged as the factors associated with the most significant variations in stress levels. Physical activity levels also showed significant differences, although these were limited to specific dimensions of the instrument (Table 2).

**Table 2:** Stress differences across the dimensions of the academic stress questionnaire

Variable	Sexo HSP / NP *n* (%)	METs (media ± dt) HSP / NP	PSQI (average ± dt) HSP/NP	*p* value (Sex)	*p* value (PSQI)
MTDS	58 (40.3) / 86 (59.7)	3245.0 (3008.9) / 3091.5 (2402.5)	9.7 (3.6) / 8.1 (3.8)	0.042	0.008
SAO	29 (27.9) / 75 (72.1)	2747.4 (1871.4) / 3245.5 (2700.3)	9.68 (3.5) / 7.9 (3.7)	0.014	0.019
BAAP	28 (20.1) / 111 (79.9)	3392.2 (3726.0) / 3085.9 (2349.8)	10.0 (3.7) / 8.4 (3.8)	0.358	0.030
PI	63 (43.2) / 83 (56.8)	3595.5 (3170.2) / 2878.8 (2227.2)	9.1 (3.8) / 8.5 (3.8)	0.001	0.348
NSC	5 (3.5) / 138 (96.5)	2402.5 (2110.9) / 3200.2 (2666.9)	7.9 (3.6) / 8.7 (3.8)	0.639	0.547
EX	71 (49.3) / 73 (50.7)	3665.9 (3259.3) / 2755.7 (1960.4)	9.35 (3.7) / 8.2 (3.8)	0.005	0.036
LVCC	12 (8.3) / 133 (91.7)	2654.3 (1636.3) / 3195.6 (2706.1)	7.6 (5.0) / 8.8 (3.7)	0.472	0.298
PD	72 (49.3) / 74 (50.7)	2812.3 (2432.2) / 3472.9 (2781.4)	9.2 (4.0) / 8.2 (3.7)	1.000	0.096

*MTDS *Methodological Deficiencies of the Teaching Staff, *SAO *Student Academic Overload, *BAAP *Beliefs About Academic Performance, *PI *Public Interventions, *NSC *Negative Social Climate, *EX *Exams, *LVCC *Lack of Value in Course Content, *PD *Participation Difficulties. ** *HSP *High Stress Potential, *NP *No Potential

Conversely, factors such as a history of mental illness and the presence of social support did not show statistically significant associations with the stress dimensions in this sample. 

### Methodological deficiencies of teachers

Significant gender differences were observed, with 40.3% of female students reporting high stress levels in this dimension (*p* = 0.042). Additionally, poorer sleep quality was significantly associated with higher stress levels in this domain (*p* = 0.008).

### Academic overload of students

The perceived workload was significantly higher among female students (*p* = 0.014). Both higher physical activity levels and better sleep quality were significantly associated with lower stress scores in this dimension (*p* = 0.019).

### Public interventions

Significant gender differences were found regarding public speaking commitments, with female students reporting higher stress levels (*p* = 0.001). No significant differences were observed regarding physical activity levels for this factor.

### Exams

Exams was perceived as a significant source of stress, particularly among female students (*p* = 0.005). Students with lower levels of physical activity reported greater stress when facing exams (*p* = 0.037). Similarly, poor sleep quality (*p* = 0.036) was associated with higher stress scores in this dimension.

### Difficulties in participation

Regardless of physical activity, women reported greater difficulty participating in academic activities compared to men (*p* = 0.001). Individuals in the high-stress group also exhibited poorer sleep quality (*p* = 0.066), although this result was on the border of statistical significance.

### Logistic regression analysis

A logistic regression analysis was performed to evaluate the association between study variables and the different dimensions of academic stress (Table 3).

**Table 3:** Multivariate logistic regression analysis of factors associated with high academic stress dimensions

Dimensions	Female Sex (Ref: Male)	Sleep Quality (PSQI Score)	Physical Activity (METs)
	aOR (95% CI)	aOR (95% CI)	aOR (95% CI)
MTDS	2.33 (1.01–5.35)*	1.12 (1.03–1.22)**	—
SAO	5.56 (1.22–25.33)*	1.14 (1.02–1.28)*	—
PI	3.02 (1.38–6.60)**	—	—
BAAP	—	—	—
EX	3.57 (1.47–8.64)**	1.12 (1.02–1.22)*	1.00 (1.00–1.00)*
NSC	—	—	—
LVCC	—	—	—
PD	—	—	—

*aOR* Adjusted Odds Ratio, *CI *Confidence Interval, *Ref *Reference category. Dashes (—) indicate variables that did not reach statistical significance in the multivariate model. *MTDS* Methodological Deficiencies of the Teaching Staff, *SAO *Students Academic Overload, *PI *Public Interventions, *BAAP *Beliefs About Academic Performance, *EX *Exams, *NSC *Negative Social Climate, *LVCC *Lack of Value in Course Content, *PD *Participation Difficulties. * *p* < 0.05; ** *p* < 0.01

### Academic overload

This factor was identified as the most prominent stressor. Women perceiving it as 5.56 times more likely to perceive academic overload as a high stressor compared to men (OR = 5.56; 95% CI: 1.22–25.33). Furthermore, for each additional point on the PSQI scale (indicating worse sleep quality), the likelihood of perceiving high stress due to overload increased by 14% (OR = 1.14; 95% CI: 1.02–1.28).

### Methodological deficiencies of teachers

Women were 2.33 times more likely to perceive methodological deficiencies as significant stressor compared to men (OR = 2.33, 95% CI: 1.01–5.35). Poorer sleep quality was also associated with this stressor, with a 12% increase in the likelihood for each unit increase in the PSQI score (OR = 1.12, 95% CI: 1.03–1.22).

### Beliefs about academic performance

A significant association was found between sleep quality and stress related to performance beliefs, showing a 12% increase in the likelihood of high stress for each additional point on the PSQI (OR = 1.12, 95% CI: 1.01–1.23).

### Public interventions

This stressor was perceived 3.02 times more frequently as significant by women compared to men (OR = 3.02, 95% CI: 1.38–6.60).

### Exams

Women are 3.57 times more likely to perceive exams as a major stressor compared to men (OR = 3.57, 95% CI: 1.47–8.64). A significant association was also observed with sleep quality (OR = 1.12, 95% CI: 1.02–1.22). In the multivariate model, physical activity did not maintain statistical significance regarding exam-related stress (OR = 1.00).

## Discussion

This study assessed perceived stress levels among medical students at the University of Cadiz, focusing on physical activity and sleep hygiene as factors associated with academic performance and concentration. The results revealed that women reported higher stress levels than their male peers, particularly in response to perceived methodological deficiencies in teaching, managing multiple academic responsibilities, and handling assessments or public presentations. This finding is consistent with previous studies that identify differential patterns of stress perception in university environments with a high academic load [22]. Rather than implying inherent fragility, these differences may reflect the persistence of social role expectations that place a higher demand on female students regarding multitasking and emotional management. Consequently, the need to incorporate an explicit gender approach in the design of curricula and in the emotional support offered by universities remains a priority. One possible explanation lies in the composition of the sample, with 78% female participation, which could reflect gender-specific stressors, such as perceived academic pressure or societal expectations. Therefore, psychological support programs should include gender-sensitive stress management tools, such as differentiated mentoring and self-care spaces tailored to the specific challenges faced by female medical students.

A crucial context for interpreting these results is the timing of data collection, which took place during a regular academic period free of final examinations. Consequently, our findings reflect a “baseline” of chronic academic overload embedded in the daily routine of the medical curriculum, distinct from the acute performance anxiety characteristic of evaluation periods.

Another key factor identified in this study was sleep quality, which showed an inverse relationship with perceived stress levels. Our results indicate that poor sleep quality is associated with higher scores in academic stress and may complicate the preparation for assessment situations. This association is consistent with international studies that have documented that sleep hygiene is a significant correlate of academic performance and emotional well-being [23, 24]. Our results are consistent with recent studies in medical students, where poor sleep quality was linked to a higher prevalence of depressive symptoms and academic stress [25, 26]. This relationship is further supported by recent multivariate analyses indicating that sleep quality acts as a critical factor between stress and academic outcomes [27]. In the context of medical education, these data provide evidence to include sleep education programs within the curricula, with practical recommendations such as establishing rest routines and limiting the use of electronic devices before bed.

Along the same lines, physical activity appears as a relevant factor related to stress. Our study found that students who engaged in more hours of physical activity experienced significantly lower stress levels. These results are consistent with previous research showing an inverse relationship between regular physical activity and perceived stress levels [28, 29]. Likewise, recent studies have highlighted that incorporating physical activity on university campuses is associated with better physical health, but also optimizes students’ academic performance and emotional resilience [30, 31].

However, as documented by Décamps et al. (2012), it is important to consider time planning, since sports overload in periods of high academic demand could potentially reverse the expected benefit and become an additional stressor [32]. Our multivariate analysis supports this, showing that when sleep is compromised physical activity loses its statistical significance regarding exam stress. This suggests that universities should create supervised exercise programmes tailored to academic cycles (e.g. by stepping up the offer of relaxation and low-impact activities during exam weeks).

Finally, family social support showed a clearly protective association. In our study, 91.7% of students feel supported by their families. This finding reinforces theories of academic resilience that highlight social support as a key resource [33]. Therefore, the implementation of formal support networks in the university environment, such as peer mentoring programs and psychological support groups, can be a high-impact intervention criterion.

### Limitations and recommendations for future research

The interpretation of these findings must be considered within the context of the study´s limitations.

The primary constraint is the cross-sectional design, which precludes establishing causal relationships between stress, sleep, and physical activity. Therefore, while associations are strong, the directionality cannot be confirmed (e.g., whether poor sleep causes stress or vice versa).

Regarding the sample, the main limitation lies in the small sample size per academic year and the voluntary nature of the survey. This may have introduced a self-selection bias, as students experiencing higher levels of stress might have been more motivated to participate than those who are less stressed, potentially overestimating the prevalence figures.

In addition, the exclusion of students from other fields of study and of Erasmus participants was necessary to ensure cultural and linguistic homogeneity, but this limits the generalizability of results. Specifically, the study was conducted exclusively with medical students. While this allows for insights into this high-demanding curriculum, results may not be generalizable to other health science degrees.

The geographical scope is also limited to the University of Cadiz, which may not reflect broader national trends. Furthermore, the lack of a longitudinal analysis prevents assessing how stress levels and coping mechanisms evolve over the academic cycle. Future research should aim to include students who engage in risky behaviors to improve academic performance, such as the use of amphetamines, caffeine, energy drinks, and cannabis [34, 35].

The current study also does not explore other relevant behavioral patterns that could influence stress levels, such as emotional balance or procrastination. Diotaiuti et al. (2021) highlight the importance of emotional balance as a mediator between procrastination and academic results, which could provide valuable information for future research [36].

To overcome these limitations, future studies should aim to encompass a wider range of university programs, include a more diverse student population, and investigate a broader range of psychosocial variables. This approach would improve understanding of the interaction between academic stress, health-risk behaviors, and sleep quality in various educational contexts.

## Conclusions

In conclusion, these results reinforce the need to promote healthy sleep habits among medical students, who face demanding study schedules and clinical rotations. Secondly, while physical activity is beneficial, our findings suggest that sleep quality is a stronger factor associated with stress resilience. Thus, prioritizing sleep hygiene is critical before recommending intense physical activity, as poor sleep may negate the potential benefits of exercise. Maintaining mental health and emotional well-being is essential not only for academic success but also for long-term professional resilience.

Furthermore, the observed gender differences suggest that specific interventions should be developed to support women in their academic stress management, as they report higher stress perception in areas such as Academic Overload and Exams. Therefore, implementing university programs focused on sleep hygiene and gender-sensitive stress management strategies could be a highly effective preventive strategy for the different academic years.

## Supplementary Information

Supplementary Material 1.

## Data Availability

The datasets generated and/or analyzed during the current study are not publicly available due to participant privacy restrictions but are available from the corresponding author on reasonable request.
