# Supplementary material for: Association between psychoeducational factors and perceived academic stress in medical students: a gender-based analysis
Source: BMC Med Educ. 2026 Feb 10;26:418. doi: 10.1186/s12909-026-08770-2 (PMC12990632; doi:10.1186/s12909-026-08770-2)
Supplement: Supplementary file 1 — Supplementary Material 1. [file 12909_2026_8770_MOESM1_ESM.pdf]

UCA Universidad de Cádiz

Comité de Ética de Experimentación no Biomédica y con Organismos Modificados Genéticamente

Vicerrectorado de Investigación y Transferencia
Centro de Transferencia Empresarial "El Olivillo"
Avda. Duque de Nájera 14
Cádiz 11002

Informe del Comité de Ética de Experimentación no Biomédica y con Organismos Modificados Genéticamente (CEENB-OMGs) de la Universidad de Cádiz.

El CEENB-OMGs informa sobre la solicitud 010_2023, presentada por D. Horacio López Ruiz, que tras haber realizado las modificaciones solicitadas y haber presentado la información requerida respecto de su solicitud inicial, el dictamen del CEENB-OMGs en su sesión de 29 de abril de 2024, es favorable.

Para que así conste y surta los efectos oportunos

Firmo este informe a fecha de 16 de mayo de 2024.

LOPEZ LOPEZ JOSE ANTONIO - 48899474W

Firmado digitalmente por LOPEZ LOPEZ JOSE ANTONIO - 48899474W
Nombre de reconocimiento (DN): c=ES, serialNumber=IDCES-48899474W, givenName=JOSE ANTONIO, sn=LOPEZ LOPEZ, cn=LOPEZ LOPEZ JOSE ANTONIO - 48899474W
Fecha: 2024.05.16 15:06:50 +02'00'

José Antonio López López
Secretario del CEENB-OMGs de la UCA
Director General de Infraestructura Científica e Institutos de Investigación
